# CT-Guided Biopsy in Suspected Spondylodiscitis – The Association of Paravertebral Inflammation with Microbial Pathogen Detection

**DOI:** 10.1371/journal.pone.0146399

**Published:** 2016-01-04

**Authors:** Daniel Spira, Thomas Germann, Burkhard Lehner, Stefan Hemmer, Michael Akbar, Jessica Jesser, Marc-André Weber, Christoph Rehnitz

**Affiliations:** 1 Diagnostic and Interventional Radiology, University Hospital Heidelberg, Im Neuenheimer Feld 110, 69120 Heidelberg, Germany; 2 Clinic for Orthopedics and Trauma Surgery, University Hospital Heidelberg, Schlierbacher Landstrasse 200a, 69118 Heidelberg, Germany; 3 Department of Neuroradiology, University Hospital Heidelberg, Im Neuenheimer Feld 400, 69120 Heidelberg, Germany; Queen Mary Hospital, HONG KONG

## Abstract

**Objectives:**

To search for imaging characteristics distinguishing patients with successful from those with futile microbiological pathogen detection by CT-guided biopsy in suspected spondylodiscitis.

**Methods:**

34 consecutive patients with suspected spondylodiscitis underwent CT-guided biopsy for pathogen detection. MR-images were assessed for inflammatory infiltration of disks, adjacent vertebrae, epidural and paravertebral space. CT-images were reviewed for arrosion of adjacent end plates and reduced disk height. Biopsy samples were sent for microbiological examination in 34/34 patients, and for additional histological analysis in 28/34 patients.

**Results:**

Paravertebral infiltration was present in all 10/10 patients with positive microbiology and occurred in only 12/24 patients with negative microbiology, resulting in a sensitivity of 100% and a specificity of 50% for pathogen detection. Despite its limited sensitivities, epidural infiltration and paravertebral abscesses showed considerably higher specificities of 83.3% and 90.9%, respectively. Paravertebral infiltration was more extensive in patients with positive as compared to negative microbiology (p = 0.002). Even though sensitivities for pathogen detection were also high in case of vertebral and disk infiltration, or end plate arrosion, specificities remained below 10%.

**Conclusions:**

Inflammatory infiltration of the paravertebral space indicated successful pathogen detection by CT-guided biopsy. Specificity was increased by the additional occurrence of epidural infiltration or paravertebral abscesses.

## Introduction

Septic spondylodiscitis is an infection involving the vertebral endplates and the intervertebral disks predisposing patients toward serious neurologic deficits, disabling pain, and sometimes death. Paravertebral and epidural tissue as well as the posterior elements of the spine can all be infested. Even with contemporary MR imaging modalities diagnosis is often not straightforward due to morphologic mimicry by degenerative, inflammatory or malignant spinal diseases, such as Modic type 1 degeneration, acute cartilaginous nodes, inflammatory or hemodialysis-associated spondylarthropathies, SAPHO/CRMO, neuropathic arthropathy, myeloma, or chordoma [1]. Even with unequivocal clinical and laboratory signs of infection and characteristic MR imaging features, the identification of the causative microbial pathogen is often requested and CT-guided biopsy can be performed to tailor antibiotic therapy. Recognition of the microbial agent is especially important in cases of atypical pathogens, such as multi-resistant agents or tuberculous infection. However, CT-guided biopsy of the spine is not a trivial procedure and may result in complications such as pain, paresis or hematoma formation [2,3]. Furthermore, it often fails to provide the pathogenic organism with variable bioptic yield. Michel et al. described successful isolation of the microbial agent in only 26.8% of patients biopsied [4], whereas Bontoux et al. and Rieneck et al. reported depiction of the microbial agent in 47.5%, and 57.1% of patients, respectively [5,6]. Therefore, imaging parameters predicting isolation of microbiological pathogens in suspected spondylodiscitis are needed to reduce unsuccessful bioptic attempts.

Here we set out to search for imaging characteristics distinguishing patients with successful from those with futile microbiological pathogen detection by CT-guided biopsy in the clinical setting of suspected spondylodiscitis.

## Materials and Methods

### Population and clinical history

This retrospective study was approved by the Ethics Committee of the medical faculty at Heidelberg University (vote S-581/2014). A search of our institution’s electronic medical record database from January 2013 to June 2015 was performed for all patients who underwent CT-guided biopsy in case of suspected spondylodiscitis. 34 consecutive patients (13 women, 21 men) with clinical and morphologic findings suggestive of spondylodiscitis underwent CT-guided biopsy for pathogen detection. Written informed consent was obtained from the patient or a guardian. In 28 of the 34 patients additional material for histological analysis was obtained. Mean age was 57.5 years (range 7–80 years). The thoracic spine was affected in 11 / 34 patients and the lumbar spine was involved in 23 / 34 patients. Underlying conditions included: none identified (n = 7), diabetes mellitus (n = 5), intravenous drug abuse (n = 1), non-spinal infection ± sepsis (n = 8), renal failure (n = 2), renal transplantation (n = 1), myeloma, pharyngeal, rectal, or breast cancer (n = 5), chronic obstructive pulmonary disease (n = 3), hypothyroidism (n = 3), liver cirrhosis (n = 1). In case of antibiotic pre-treatment (n = 5), antibiotic medication was stopped at least 7 days before CT-guided biopsy to enable pathogen detection.

### Imaging Evaluation

#### Imaging technique

All 34 patients received a pre-interventional contrast-enhanced MRI of the spine and an additional CT for biopsy planning. MR imaging was performed with a 3.0 T system (Magnetom Verio, Siemens Healthcare, Erlangen, Germany). T1-weighted turbo spin-echo sequences were obtained with 700–800 ms TR, 12 ms TE, flip angle 150–180°, and an echo train-length of three. T2-weighted turbo spin-echo sequences were obtained by using 2.2–7.9/113-119 ms TR/TE, flip angle 150–180°, and an echo train-length of 19. Short tau inversion recovery sequences were obtained with 5.1–8.9 ms TR, 44–45 ms TE, 210 ms inversion time, flip angle 150–180°, and an echo train-length of 19. Contrast-enhanced T1-weighted turbo spin-echo sequences were obtained by using 700-1000/11-12 ms TR/TE, flip angle 150–180°, and an echo train-length of three. Gadoterate meglumine (Dotarem; Guerbet, Roissy, France) at a dose of 0.1 mmol per kg body weight was used as intravenous contrast agent.

#### Imaging findings

MR imaging findings suggesting infection were defined as edema or contrast-enhancement of the intervertebral disk, adjacent vertebrae, epidural and paravertebral space, or the occurrence of abscesses. Signal intensities (SI) were compared to normal adjacent disks, vertebrae, epidural and paraspinal soft tissue and were graded as ***edematous*** when increased on short tau inversion recovery (STIR) sequences. Analogously, the degree of ***contrast enhancement*** was compared and decided to be increased when elevated on contrast-enhanced T1-weighted fat saturated spin echo sequences. An ***abscess*** was defined as iso- or hypointensity compared to muscle tissue on T1-weighted images, fluid-equivalent SI on T2-weighted images and rim enhancement on contrast-enhanced T1-weighted fat saturated images [7]. CT images were reviewed for ***arrosion*** of adjoining end plates (defined as cortical irregularity or defect) and ***reduced disk height*** (as compared to neighboring disks). To verify investigator observations in an unbiased manner, an independent evaluation of every MRI and CT scan was performed retrospectively by two radiologists (DS and CR). Both readers were blinded to laboratory results and clinical response. Discrepant results were solved by consecutive consensus reading.

### CT-guided biopsy

The vertebral level and interventional approach was defined according to pre-interventional MRI. All patients received moderate IV sedation using 15 mg piritramid (Dipidolor, Hameln, Germany) and 1.25 mg– 2.5 mg midazolam (Dormicum, Hameln, Germany) with continuous monitoring of vital signs. CT-guided biopsy was performed on a Somatom Emotion scanner (Siemens Medical Solutions). A thin-slice planning CT scan (0.75 mm slice thickness) was done in prone position and multiplanar reconstructions were used to non-traumatically position the biopsy needle. Aseptic preparation of the skin overlying the biopsy needle trajectory and consecutive local anesthesia with 5 ml Carbostesin 0.5% (Astra Zeneca, Wedel, Germany) was administered. A small skin incision was made and biopsy was performed using a 3.0 mm (11 gauge) or 4.0 mm (8 gauge) biopsy system (Bone Marrow Biopsy Needle, Somatex, Germany or Bard Coaxial Biopsy Needle, Bard Peripheral Vascular, Inc., USA). A transpedicular approach was chosen in 26 / 34 patients and a posterolateral approach was chosen in 8 / 34 patients. When abscesses were biopsied additional aspiration of liquefied content was performed. A biopsy sample was sent for microbiological examination in all 34 / 34 patients, and an additional sample was formalin-fixated for histological analysis in 28 / 34 patients.

### Statistics

All data are reported as arithmetic mean ± S.D. or range, as appropriate. We calculated the sensitivity, specificity, accuracy, positive predictive value (PPV) and negative predictive value (NPV) for each of the different MR imaging signs described above. Statistical analysis was performed with Stata 10.0 (StataCorp LP). Shapiro-Wilk test revealed a non-normal distribution of our data. Therefore, the Mann-Whitney *U* test was used for comparisons between groups. For all tests *p* values ≤ 0.05 were considered as statistically significant.

## Results

### Microbiology and histology

Causative pathogens were detected in 10 / 34 patients, including Enterobacteria (n = 2), staphylococci (n = 4, one being methicillin-resistant), Aggregatibacter aphrophilus (n = 1), Mycobacterium tuberculosis (n = 2), and Escherichia coli (n = 1). A causative fungal agent was detected in none of the biopsy samples. Histological analysis revealed signs of inflammation in 21 / 28 patients, and showed an unspecific, non-inflammatory cellular composition in 7 / 28 patients. 6 patients were referred solely for microbial pathogen detection. Malignancy was excluded in all patients by either non-malignant histology (n = 28) or improvement of symptoms and inflammatory changes on follow-up MRI (n = 6).

### Soft tissue inflammation versus microbiology & histology

**Fig 1:**
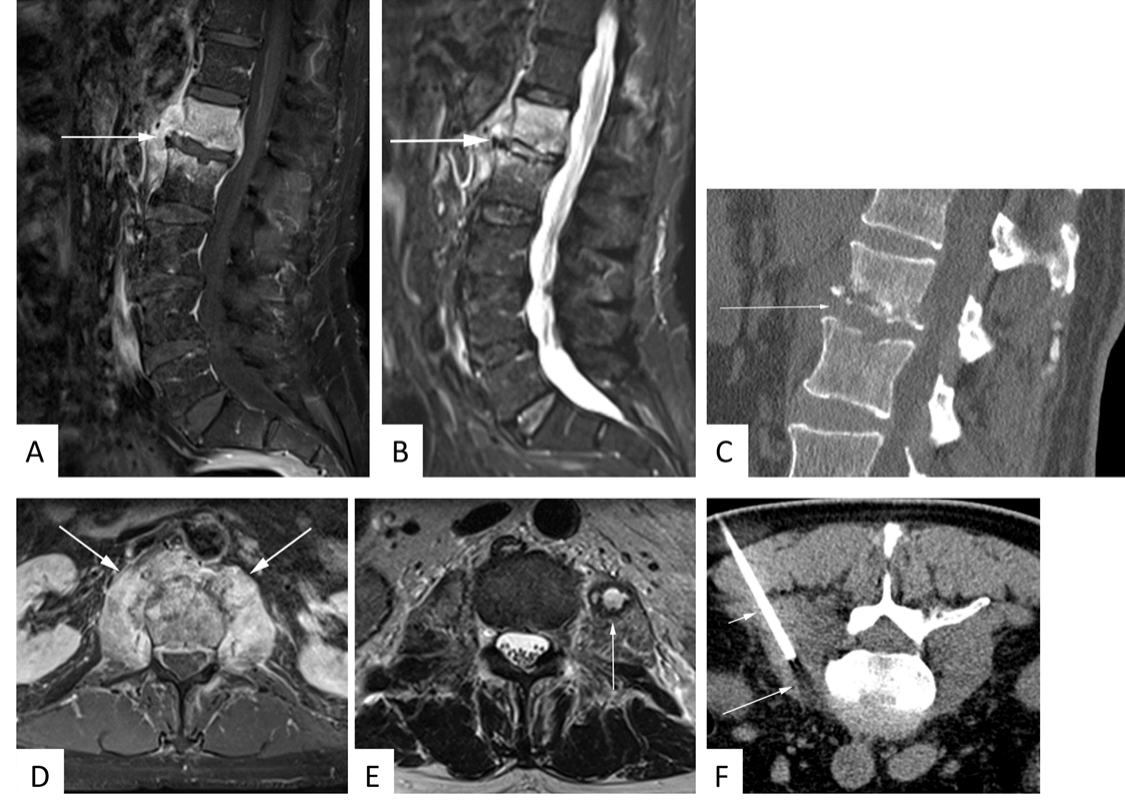
63 year-old male patient with suspected spondylodiscitis. Sagittal T1-weighted fat-saturated contrast-enhanced sequence **(A)** demonstrates florid enhancement of adjacent vertebral bodies and paravertebral soft tissue. Note concomitant T2-hyperintensity of the disk space as well as vertebral and paravertebral edema on sagittal STIR image **(B)**. Sagittal CT reconstructions reveal arrosion of adjoining end-plates **(C)**. Paravertebral soft tissue enhancement is even more conspicuous on contrast-enhanced axial T1-weighted fat-saturated sequence **(D)**. An abscess is identified in the left psoas muscle as T2-hyperintensity surrounded by a T2-hypointense rim **(E)**. CT-guided biopsy of the psoas abscess disclosed methicillin-resistant staphylococcus aureus infection **(F)**.

**Fig 2:**
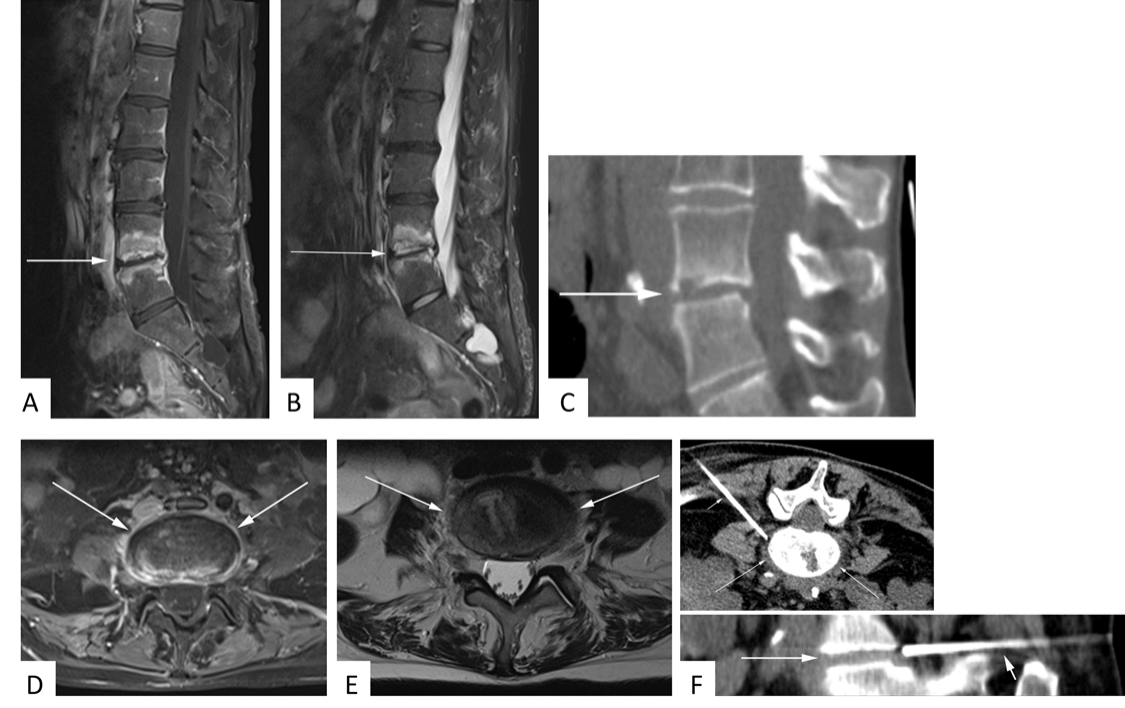
53 year-old female patient with suspected spondylodiscitis. Sagittal T1-weighted fat-saturated contrast-enhanced sequence **(A)** again illustrates prominent enhancement of adjacent vertebral bodies but not of paravertebral soft tissue. T2-hyperintensity of the disk space and vertebral edema is documented on sagittal STIR image **(B)**. Sagittal CT reconstructions illustrate arrosion of the bottom end-plate L4 **(C)**. No significant paravertebral soft tissue infiltration is appreciated on contrast-enhanced axial T1-weighted fat-saturated **(D)** or axial T2-weighted **(E)** sequences. 2.2 cm CT-guided core biopsies of the intervertebral space from the documented position failed to provide a causative microbiological pathogen **(F)**.

***Paravertebral infiltration*** was present in all 10 / 10 patients with positive microbiology and occurred in only 12 / 24 patients with negative microbiology (Table 1) (Figs 1 and 2). Hence, with a sensitivity of 100% paravertebral infiltration reliably indicated successful pathogen detection, holding a specificity of 50% (Table 2). ***Epidural infiltration*** was observed in 8 / 34 patients and did not occur in the absence of an inflammatory histology. Even though its sensitivity was limited (40.0%) it showed a considerably higher specificity of 83.3% for pathogen detection (Tables 1 and 2). ***Paravertebral abscesses*** accompanied paravertebral infiltration in 6 / 34 patients and came along with an even higher specificity of 90.9% for successful pathogen detection (Table 2).

**Table 1:** Soft tissue inflammation versus microbiology & histology.

Soft tissue inflammation	Pathogen detection		Inflammatory histology	
	Yes (n = 10)	No (n = 24)	Yes (n = 21)	No (n = 7)
Paravertebral infiltration	10 / 10	12 / 24	15 / 21	2 / 7
Epidural infiltration	4 / 10	4 / 24	6 / 21	0 / 7
Paravertebral abscess	4 / 10	2 / 24	4 / 21	0 / 7
Paravertebral infiltration > 5 mm	10 / 10	9 / 24	14 / 21	2 / 7

Soft tissue inflammation was categorized into paravertebral infiltration, epidural infiltration, paravertebral abscess, and paravertebral infiltration > 5 mm. Patients with/without successful pathogen detection and with/without inflammatory histology are specified.

**Table 2:** Performance of soft tissue inflammation in pathogen detection.

Soft tissue inflammation	Sens [%]	Spec [%]	Acc [%]	PPV [%]	NPV [%]
Paravertebral infiltration	100.0	50.0	64.7	45.5	100.0
Epidural infiltration	40.0	83.3	70.6	50.0	76.9
Paravertebral abscess	40.0	90.9	75.0	66.7	76.9
Paravertebral infiltration >5 mm	100.0	62.5	73.5	52.6	100.0

Soft tissue inflammation was categorized into paravertebral infiltration, epidural infiltration, paravertebral abscess, and paravertebral infiltration > 5 mm. Sensitivities, specificities, accuracies, PPVs, and NPVs for successful pathogen detection by CT-guided biopsy are specified.

The extent of paravertebral infiltration was larger in patients with positive (n = 10) as compared to negative (n = 12) microbiology (p = 0.002) (Table 3). Mean values of paravertebral abscesses were higher in patients with positive (n = 4) as compared to negative (n = 2) microbiology, however not statistically significant due to the small sample size (p>0.05). Likewise, as only 2 patients with documented non-inflammatory histology showed paravertebral inflammation and none of those presented with abscesses, the higher means of inflammatory (as compared to non-inflammatory) histology did not reach statistical significance (p>0.05) (Table 3). Nevertheless, it seemed likely that adding a threshold for the extent of ***paravertebral infiltration*** would help to increase its moderate specificity of 50%. The lowest diameter of paravertebral soft tissue infiltration in patients with positive microbiology was 8 mm. When choosing a ***paravertebral infiltration > 5 mm*** as threshold, all 10 / 10 patients with microbiological pathogen detection were recognized and only 9 / 24 patients would have been biopsied with negative microbiology (Table 1). With a sensitivity and NPV of 100%, respectively, paravertebral soft tissue infiltration > 5 mm reliably indicated successful pathogen detection, holding a specificity of 62.5% (Table 2).

**Table 3:** Extent of paravertebral inflammation versus microbiology & histology.

Paravertebral inflammation	Pathogen detection		Inflammatory histology	
[mm]	Yes (n = 10)	No (n = 24)	Yes (n = 21)	No (n = 7)
Paravertebral infiltration (n = 22)	21.9 ± 14.1	8.3 ± 4.4	14.9 ± 12.4	6.5 ± 0.7
Paravertebral abscess (n = 6)	21.3 ± 11.4	8.0 ± 2.8	13.3 ± 6.9	-

In the 22 patients with paravertebral infiltration and in the 6 patients with paravertebral abscesses, the width of paravertebral infiltration and diameters of paravertebral abscesses were measured on contrast-enhanced axial T1-weighted fat-saturated MRI sequences. Mean values ± SD of patients with/without successful pathogen detection and with/without inflammatory histology are specified.

### Vertebral body & disk space inflammation versus microbiology & histology

Even though the sensitivities for pathogen detection were also high in case of ***vertebral*** and ***disk infiltration***, or ***end plate arrosion***, specificities remained below 10% with all three parameters (Tables 4 and 5). This is because edema or enhancement of the disk and adjacent vertebrae, as well as arrosion of the adjoining end plates were present in almost all patients and did not distinguish patients with successful pathogen detection or inflammatory histology from those with negative microbiology or unspecific histology (Tables 4 and 5). ***Reduced disk height*** was neither sensitive nor specific for microbiological or histological confirmation of disease (Tables 4 and 5).

**Table 4:** Vertebral body & disk space inflammation versus microbiology & histology.

Vertebral body & disk space	Pathogen detection		Inflammatory histology	
	Yes (n = 10)	No (n = 24)	Yes (n = 21)	No (n = 7)
Vertebral infiltration	10 / 10	23 / 24	21 / 21	6 / 7
End plate arrosion	9 / 10	22 / 24	19 / 21	7 / 7
Disk infiltration	10 / 10	22 / 24	19 / 21	6 / 7
Disk height reduced	2 / 10	13 / 24	8 / 21	3 / 7

Imaging findings were categorized into vertebral infiltration, end plate arrosion, disk infiltration, and reduced disk height. Patients with/without successful pathogen detection and with/without inflammatory histology are specified.

**Table 5:** Performance of vertebral body & disk space inflammation in pathogen detection.

Vertebral body & disk space	Sens [%]	Spec [%]	Acc [%]	PPV [%]	NPV [%]
Vertebral infiltration	100.0	4.2	32.4	30.3	100.0
End plate arrosion	90.0	8.3	32.3	29.0	66.7
Disk infiltration	100.0	8.3	35.3	31.3	100.0
Disk height reduced	20.0	45.8	38.2	13.3	57.9

Imaging findings were categorized into vertebral infiltration, end plate arrosion, disk infiltration, and reduced disk height. Sensitivities, specificities, accuracies, PPVs, and NPVs for successful pathogen detection by CT-guided biopsy are specified.

## Discussion

Imaging characteristics of septic spondylodiscitis have been investigated extensively and the high prevalence of paravertebral inflammation in spinal infections has been described by several groups [8–11]. This study shows that inflammatory infiltration of the paravertebral space in patients with clinical and MR imaging findings suggestive of spondylodiscitis reliably indicates successful pathogen detection by CT-guided biopsy.

The tendency of spinal infections to infest paraspinal soft tissue goes along nicely with the typical anatomy of the paravertebral venous plexus, which does not contain valves and allows antegrade and retrograde blood flow [12]. Thus, pathogens may reach the spine from the abdomen and pelvis causing spinal disease, but are also likely to secondarily involve paravertebral soft tissue in case of spinal infections. In our cohort, paravertebral infiltration disclosed all 10/10 patients with positive microbiology and occurred in only 12/24 patients with negative microbiology. Also, paravertebral infiltration itself was more extensive in patients with positive as compared to negative microbiology. Hence, even a threshold of paravertebral soft tissue infiltration > 5 mm reliably indicated successful pathogen detection (100% sensitivity, 62.5% specificity, 100% NPV). Furthermore, Stäbler and Reiser described paravertebral and epidural abscesses as important features distinguishing pyogenic spondylodiscitis from erosive intervertebral osteochondrosis or erosive degenerative disk disease [13]. In case of epidural infiltration, the inflammation initially remains confined to the space beneath the posterior longitudinal ligament, resulting in a characteristic tent-shape appearance [14–16]. In our patient cohort, the occurrence of either epidural infiltration or paravertebral abscesses showed high specificities for microbiological pathogen detection after CT-guided biopsy (83.3% and 90.9%, respectively). Therefore, CT-guided biopsy proved to be particularly effective in the presence of epidural infiltration or paravertebral abscesses and should be strongly recommended in this clinical scenario.

Edema or enhancement of the disk space and adjacent vertebrae, as well as arrosion of the adjoining end plates are well recognized imaging characteristics of infectious spinal disorders [11,17,18]. They were observed in almost all of our patients with suspected spondylodiscitis, conforming to high sensitivities but very low specificities for bioptic pathogen detection. Some authors describe disk height to be decreased in patients with disk infections [8,19], whereas Ledermann et al. report normal disk height in more than one third of patients [11]. In our patient cohort, reduced disk height was neither sensitive nor specific for bioptic confirmation of disease. Table 6 therefore suggests an imaging-guided algorithm in clinically suspected spondylodiscitis tailoring CT-guided biopsy to those patients in whom successful pathogen detection can be expected.

**Table 6:** Imaging-guided algorithm in clinically suspected spondylodiscitis.

Paravertebral abscess or epidural infiltration	Paravertebral infiltration	Inflammatory changes confined to vertebra and disk space
Highly specific (high diagnostic yield)	Highly sensitive	Unspecific
CT-guided biopsy strongly recommended	CT-guided biopsy should be considered	MRI follow-up according to clinical disease course (except in case of immunosuppression*)

Imaging findings were categorized into “Paravertebral abscess or epidural infiltration”, “Paravertebral infiltration”, and “Inflammatory changes confined to vertebra and disk space”. Recommendations for successful pathogen detection by CT-guided biopsy are specified. *In case of immunosuppression a CT-guided biopsy should be considered for early detection of opportunistic pathogens (such as fungal infections)

Empirical broad-spectrum antibiotic therapy was linked to increased resistance, post-antibiotic complications (e.g. Clostridium difficile-associated diarrhea) and health care costs [20,21]. As multi-resistant and atypical agents are increasingly recognized, identification of the microbial pathogen is needed for sensitivity testing and to tailor antibiotic therapy [20]. In our study, 30% of causative pathogens isolated turned out to be atypical (i.e. methicillin-resistant staphylococcus aureus and tuberculous infections). This highlights the importance to correctly determine the microbial agent, whenever possible.

There are some limitations that need to be discussed. Firstly, additional histological analysis was only performed in 28/34 patients. However, this was not the principal point of our study, which focused on pathogen detection in suspected spondylodiscitis. Furthermore, malignancy was excluded in all patients by either non-malignant histology or improvement of symptoms and inflammatory changes on follow-up MRI. Secondly, due to ethical concerns and the retrospective nature of our analysis the exact number of false-negative biopsies remains elusive, as no gold-standard superior to CT-guided biopsy (such as post-mortem studies or open surgical resection) was performed. Our goal was not to assess the accuracy of CT-guided biopsy for pathogen detection but to assist the clinician when confronted with the question whether or not to submit her patient for CT-guided biopsy when searching for the microbiological agent in the scenario of suspected spondylodiscitis. Thirdly, none of the biopsy samples revealed a causative fungal agent. This is easily explained by the rare occurrence of fungal spondylodiscitis, even in large series, and its strong association with immunosuppression [22]. The lack of fungal pathogens in our cohort limits the generalization of our results to some degree and we would therefore still advocate biopsy in strongly immunosuppressed patients even in the abscence of paravertebral inflammation, in order to not overlook a fungal culprit.

Besides CT-guided core biopsy, fine-needle aspiration and surgical biopsy are used [4,23,24]. However, all those techniques are compromised by false negative results which may be due to inadequate sampling, antibiotic therapy administered before biopsy, or insufficient numbers of infectious organisms within the biopsy tissue [4,23,24]. Therefore, imaging parameters predicting successful isolation of microbiological pathogens in suspected spondylodiscitis are needed to reduce unnecessary bioptic attempts.

In conclusion, we show that inflammatory infiltration of the paravertebral space indicates successful pathogen detection by CT-guided biopsy. Specificity is increased by the additional occurrence of epidural infiltration or paravertebral abscesses.
